# The risks of artificial intelligence: A narrative review and ethical reflection from an Oral Medicine group

**DOI:** 10.1111/odi.15100

**Published:** 2024-08-23

**Authors:** Qingmei Joy Feng, Molly Harte, Barbara Carey, Ali Alqarni, Luis Monteiro, Márcio Diniz‐Freitas, Jean‐Cristophe Fricain, Giovanni Lodi, Vlaho Brailo, Mattia Andreoletti, Rui Albuquerque

**Affiliations:** ^1^ Oral Medicine Guy's and St Thomas' NHS Foundation Trust London UK; ^2^ Department of Head and Neck Surgical Oncology Guy's and St Thomas' NHS Foundation Trust London UK; ^3^ Department of Oral & Maxillofacial Surgery and Diagnostic Sciences, Faculty of Dentistry Taif University Taif Saudi Arabia; ^4^ Medicine and Oral Surgery Department University Institute of Health Sciences (IUCS), UNIPRO, CESPU Gandra Portugal; ^5^ Special Care Dentistry Unit, School of Medicine and Dentistry University Santiago de Compostela Santiago de Compostela Spain; ^6^ Oral Surgery Department University of Bordeaux Bordeaux France; ^7^ Dipartimento di Scienze Biomediche, Chirurgiche e Odontoiatriche Università Degli Studi di Milano Milan Italy; ^8^ School of Dental Medicine University of Zagreb Zagreb Croatia; ^9^ Department of Health Sciences and Technology ETH Zurich Zurich Switzerland; ^10^ Faculty of Dentistry, Oral & Craniofacial Sciences King's College London London UK

**Keywords:** artificial intelligence, dentistry, ethics

## Abstract

As a relatively new tool, the use of artificial intelligence (AI) in medicine and dentistry has the potential to significantly transform the healthcare sector. AI has already demonstrated efficacy in medical diagnosis across several specialties, used successfully to detect breast, lung and skin cancer. In Oral Medicine, AI may be applied in a similar fashion, used in the detection and diagnosis of oral cancers and oral potentially malignant diseases. Despite its promise as a transformative diagnostic aid, the use of AI in healthcare presents significant safety, reliability and ethical concerns. There is no formal consensus on the safe and ethical implementation of AI systems in healthcare settings, but the literature converges on several key principles of ethical AI use including transparency, justice and fairness, non‐maleficence, responsibility and privacy. This article provides a narrative review of the key ethical issues surrounding AI use in medicine, and reflects on these, providing view‐points of a bioethicist and Oral Medicine clinicians from several units.

## 1 INTRODUCTION

Notable progress has been made in medical diagnostics due to the rapid development in artificial intelligence (AI) in recent years. The term ‘artificial intelligence’ was first used by Professor John McCarthy in 1955 in order to explain and describe ‘the science and engineering of making intelligent machines’ (Manning, 2020). AI refers to the simulation of human intelligence processes, such as perceiving, reasoning, learning and problem‐solving, by machines and computer systems (Craig et al., 2024). AI has several subsets. Machine learning (ML) employs algorithms based on historical data to detect patterns, and thereafter makes predictions (McKinsey & Company, 2023). These algorithms adapt to new data, improving in ability with increasingly large data training sets, and can process data sets too vast for human labour to manage (McKinsey & Company, 2023). Deep learning (DL) is a type of machine learning which can process a far larger range of data. DL uses artificial neural networks which mimic neurons and synapses in the human brain. Through neural networks, data can be processed through multiple hidden layers, each new layer taking into account the output data from the previous layer, before providing a final result (McKinsey & Company, 2023). This gives DL the ability to independently assess data, learn from it and apply the new knowledge to make informed decisions about new data (McKinsey & Company, 2023).

The growing use of AI in medicine over the past two decades highlights its potential for enhancing diagnostics and treatment of disease (Nguyen et al., 2021). Technical advances in AI technologies have propelled its use in a variety of healthcare settings, with particular benefits in medical diagnostics. The potential benefits of AI in medical diagnostics are reflected by improvements in the rapid detection and treatment of breast, lung and skin cancers (Ahn et al., 2023; Gandhi et al., 2023; Melarkode et al., 2023). In dentistry, AI models have been proposed for the detection of salivary biomarkers for a number of oral and maxillofacial diseases (Adeoye & Su, 2023), as well as for the detection of dental caries on clinical photographs (Moharrami et al., 2024). Despite the potential benefits AI brings to healthcare, the introduction of any new technology to this setting poses risks and challenges, and it is important that healthcare providers introducing and using AI have a good understanding of its potential shortcomings and associated ethical issues (Vayena et al., 2018).

Although some advances have been made in diagnostic techniques for oral cancer, lack of high‐quality trial data limits the use of these novel tools (Chakraborty et al., 2019). While the integration of AI into Oral Medicine practice offers the possibility of improving early detection of oral cancer and oral potentially malignant disorders (Rokhshad et al., 2024), it also carries substantial safety, reliability and ethical concerns. Morch et al. identified 45 ethical issues related to the use of AI in dentistry (Mörch et al., 2021). Despite the growing use of AI across healthcare settings, the number of publications addressing its ethical use has remained consistent. This is reflected in both a lack of comprehensive information on the subject, and in a lack of consensus agreement on what ethical AI use looks like within healthcare.

This article provides a cautionary narrative for Oral Medicine clinicians as well as those working in other healthcare sectors, emphasising the need for careful and responsible integration of emerging AI tools into their professional practice. Our aim is to review literature published on the use of AI in various medical fields, to form a narrative review of the most pertinent ethical issues surrounding AI use. It reflects the views of a bioethicist and Oral Medicine clinicians across several units: Guy's and St Thomas's NHS Foundation Trust/King's College London (England), CESPU University (Portugal), University of Zagreb (Croatia), University of Bordeaux (France), University of Milan (Italy), TAIF University (Saudi Arabia), University of Santiago de Compostela (Spain) and ETH Zurich (Switzerland).

## 2 ETHICAL ISSUES SURROUNDING THE USE OF AI IN MEDICAL DIAGNOSIS

### 2.1 Quality of data and healthcare inequalities

The quality of training data input directly influences the quality of the resultant AI: AI algorithms learn from patterns and features present in the data they are trained on. This means that the input of large sets of data, representative of diseases, is required to achieve an AI programme capable of diagnosing diseases with high levels of sensitivity and specificity (Mahmood et al., 2020). The choice of data included for training purposes will substantially influence the AI's performance. The data selection process is not transparent and may therefore lead to the assumption that AI functions independently. However, AI is not completely independent, particularly in more advanced machine learning (ML) and deep learning (DL) systems. The decisions made by an AI system are strongly influenced by the initial training data. This necessitates an excellent training data set, with diagnoses made by an expert clinician, and photographs standardised across cases. Where the training data are limited or biased, the AI model may be incapable of diagnosing new, unseen cases. The quality of training data is also relevant when considering the use of text‐generative AI systems such as ChatGPT by OpenAI. Inconsistencies in the training data can be reflected as inaccuracies in the responses provided by such software (Santana, 2023), leading to uncertainty and potential harm in the case of outdated or incorrect responses (Diniz‐Freitas et al., 2023).

The training data, however, are rarely accessible, making it difficult for researchers to assess quality. Nonetheless, it has been repeatedly demonstrated that often there is a disproportionate representation of minority groups within health data. This imbalance arises for reasons such as lack of access to healthcare, under‐sampling of minority groups, and the exclusion of clinically relevant demographic variables from the data sets (Leslie et al., 2021). This may introduce algorithmic biases and exacerbate existing healthcare inequalities for minority groups (Leslie et al., 2021). For example, it is well established that the diagnosis of non‐melanoma skin cancer is frequently delayed in non‐white patients, which results in higher morbidity and mortality rates (Higgins et al., 2018). This phenomenon is mirrored when AI is employed in diagnosis (Willingham Jr. et al., 2021).

In addition to requiring large and representative data sets, the annotation of data is crucial in ensuring the proper functioning of AI systems. Annotation of data is a human process undertaken by medical professionals. It is tedious, liable to human error, and susceptible to intra‐ and inter‐observer bias. Any errors introduced in the data‐annotation stage results in the skewing of data, which may propagate further errors in the AI algorithms, resulting in flawed and unreliable AI (Zhang & Zhang, 2023).

### 2.2 Data ownership and privacy

Another pertinent ethical issue surrounding the use of AI in diagnostics is the ownership of data and data protection. Privacy is a major concern with the introduction of any technological healthcare aid (Andreoletti et al., 2024) and is a considerable concern with the introduction of AI in medicine. Most AI training data sets are not consolidated for the specific purpose of developing AI models but have been extracted from patients' personal health records. In Europe, the EU Commission Regulation 2016/679 states that patient data may only be reused in medical AI if the data have been completely anonymised, is part of a governmental act or patients have provided informed consent for their data to be used (Müller, 2022). The need for gaining informed consent has been associated with a disproportionate imbalance between administrative burdens and quantity of training data (Mittelstadt & Floridi, 2016; Ruyter et al., 2010). Furthermore, with regard to the use of anonymised or pseudonymised data, there is also a risk of re‐identification of data with increasingly sophisticated AI technology (Müller, 2022). The issue of protecting anonymity is compounded when dealing with rare diseases and conditions, where the data set is small: the risk of re‐identification by an advanced AI software is increased. This highlights a serious risk of sensitive information being published without patient consent, with patient information being vulnerable to such re‐identification (Packhäuser et al., 2022). This is a key issue in Oral Medicine, where rare conditions are perhaps more frequently encountered.

### 2.3 Corruption of data

AI algorithms may be susceptible to data corruption. Finlayson et al. (2019) showed that the introduction of only 4% adversarial noise to an original image could cause AI programmes to change their decision in the diagnosis of skin cancer, with a benign lesion being incorrectly diagnosed as a malignant one (Finlayson et al., 2019). These same changes in the corrupted images were not easily detected by the human eye (Finlayson et al., 2019). The susceptibility of AI to subversion may be of little interest to most at present but carries far‐reaching implications to various stakeholders considering the amount of money, technology, time and information invested in AI, as well as the potential dependence of patients on diagnoses generated by AI (Finlayson et al., 2019). Furthermore, it implies that there is a risk that any artefacts in raw data, such as radiological and histological images, which would normally be identified and accounted for by human practitioners, may be misinterpreted by AI, leading to incorrect diagnoses.

### 2.4 Black‐box decision making

Human clinicians are able to justify and rationalise the decisions they make using the three pillars of evidence‐based medicine. In AI systems, the transparency and traceability of the decision‐making process is very limited (Bagchi, 2023). This becomes a particular issue in deep learning systems, where input data are processed via an unknown number of network levels before reaching the final layer (McKinsey & Company, 2023). This is known as the ‘black box’ phenomenon, where the internal workings of an AI system remain opaque to the users and, possibly even to the developers. The system's code or reasoning that led to a certain conclusion is inaccessible for investigation (Bagchi, 2023). As a result, flaws or biases in algorithms might be difficult to identify (Challen et al., 2019), possibly leading to faulty outcomes if not inspected and regulated. As well as threatening the reliability of the AI system, the black‐box phenomenon may also reduce its credibility. The inability of an AI system to provide rationale for a decision made may also lead to frustration from clinicians and patients who are unable to understand how a particular outcome has been reached, especially in the event that the decision made does not match expectations (Zhang & Zhang, 2023). In turn, this may reduce trust in the capability of the AI model, acting as a potential barrier to the adoption of AI in routine clinical practice.

The quality of training of an AI model, as previously addressed, significantly impacts on the decisions it makes and the black‐box phenomenon, where there is a lack of transparency about how an incorrect decision was reached, then hinders the possibility of further training of the AI model to correct this in future.

### 2.5 Responsibility

Dental and Medical practitioners are held responsible for their clinical decisions: they must adhere to the criteria established by their professional registration and exhibit evidence of ongoing training and development throughout their careers. AI systems should be subject to the same scrutiny: their performance must be in line with the same professional standards their clinical counterparts are subject to, and they must be able to adapt to new information and developments within the field of medicine in which they are used. In the event of a clinical error, the extent to which AI systems are liable may be called into question: does the responsibility lie with the medical professional utilising the AI, the institution who has introduced the AI, the AI developer or an independent regulator? (Zhang & Zhang, 2023). This issue is further compounded by the previously discussed black‐box phenomenon: the opaque nature of AI algorithms could also affect clinicians' liability. When clinicians rely on AI models for patient care decisions without clear explanations, they may face serious challenges regarding their ethical and legal obligations. Clinicians have a moral and legal duty to ensure that patients are fully informed about their care and the decision‐making process that surrounds it. Due to the black‐box phenomenon, when AI models make recommendations without a clear explanation, fully informing the patient becomes more difficult, thus jeopardising patient autonomy and informed consent. In the event of adverse outcomes or medical errors, the opacity of AI‐driven decisions could complicate investigations and legal proceedings.

Furthermore, overdependence on AI's diagnostic capabilities risks the reintroduction or reinforcement of paternalistic healthcare models, where AI‐based decisions are valued over human judgement, and a shared‐decision making process is undermined (Zhang & Zhang, 2023), or, when two conflicting medical opinions are present, confusion, dissatisfaction and delays in treatment ensue, which negatively impact on patient outcomes. Where there is a conflict between AI‐based decisions and human decisions, the question then lies in which decision should take precedence. A human can justify their decision, explaining how they came to it, whereas an AI software, given the black‐box phenomenon, simply provides a decision without the insight of how it was reached.

### 2.6 Cost of AI misdiagnosis

Unlike human clinicians, AI algorithms are not trained to consider the cost and possible repercussions of an incorrect diagnosis. For example, in a comparison of AI systems and dermatologists in the diagnosis of benign and malignant melanocytic lesions, AI performed at the same level with dermatologists, but humans tended to over‐diagnose malignancy when differentiating benign and malignant lesions (Esteva et al., 2017). The human tendency to err on the side of caution comes from the understanding of the serious consequences of a misdiagnosed malignant lesion (i.e. precautionary principle), which however may result in perceived poorer statistical performance compared to AI technology. As such, the real‐world cost of false positives and negatives should be a factor in the development and training of AI systems (Megler & Gregoire, 2018).

The ethical principle of nonmaleficence: to prevent harm, is inextricably tied to the AI validation process, with clinical validation being the most critical level of validation for clinicians using AI models. Before being deployed in healthcare, AI models must go through rigorous validation methods (Park et al., 2021). Clinical validation entails assessing whether the measurement accurately represents the intended concept, such as the patient's subjective experience, functional ability or overall well‐being. It involves determining if a specific measure adequately captures relevant aspects of how a particular patient population feels, functions or survives. When considering clinical validation, it is critical to examine the possible harm that an AI model might do owing to false positives, false negatives or misinterpretation of data (Megler & Gregoire, 2018). Designing appropriate validation techniques for AI software to dependably prevent damage is a complicated and difficult undertaking.

### 2.7 Patient access to AI programmes for self‐diagnosis

AI in healthcare has so far been mostly deployed in high‐income countries, and remains relatively unused in resource‐poor settings (Wahl et al., 2018). However, recently there has been increasing use of various AI technologies in lesser economically developed countries, evidencing potential for AI to be utilised in the reduction of healthcare inequalities and improving health outcomes in these areas (Wahl et al., 2018), particularly in low‐income countries.

Examples of meaningful ways in which AI have been beneficial include the supporting of local physicians in diagnosing and managing patients with the standards and medical knowledge available in high‐income countries, or even acting in place of a human doctor if one is not available locally (Wahl et al., 2018).

Nonetheless, many barriers persist, hindering the widespread adoption of AI in lower‐income countries, some of which may be independent of economic factors. Limitations, such as the quality of audio‐visual data, or biases within data sets due to lack of representation of minority groups continue to impede AI‐based diagnosis in resource‐poor settings (Wahl et al., 2018). Compounding this is resource availability: while AI has the potential to remotely diagnose disease and suggest appropriate treatment, there is no guarantee that medical facilities in lesser economically developed countries have the available resources, technology or infrastructure required to support the recommendations made by an AI model, meaning patients may still be unable to access the appropriate or recommended treatment. This may lead to persistent healthcare inequalities (Wahl et al., 2018).

Finally, there is the problem of converting both the AI input and output data to the clinical language of healthcare services in different settings and contexts. Medical terminology is not internationally defined, and health records are likely to be preserved in a language specific to the healthcare service environment. This creates another barrier to the adoption of AI, where either the benefit that can be derived from AI is reduced, or significant administrative, clinical and academic efforts are needed to overhaul the local clinical language (Table 1).

**TABLE 1:** Summary of the key ethical issues when implementing AI systems in a healthcare setting.

Ethical issue	Key points
Quality of data and healthcare inequalities	The quality of training data provided for an AI system significantly impacts on its ability. Large, representative data sets are needed, and biases within these data sets will result in biases within the AI system
Data ownership and privacy	Patient consent and data anonymisation is a key consideration when providing training data. A risk of re‐identification of anonymised data exists, especially with respect to rare diseases
Corruption of data	AI systems are vulnerable to data corruption. Minimal adversarial noise can significantly alter an AI system's decision making
Black‐box decision making	The decision‐making processes of an AI system are often not traceable. The inability to understand why a particular decision has been made may lead to lack of trust in the AI system
Responsibility	The responsibility for decisions made by an AI system is complex and may involve the AI developer, the implementing healthcare organisation, and the clinician. The black‐box phenomenon makes it challenging to hold AI systems accountable for decisions made
Cost of AI misdiagnosis	Unlike their human counterparts, AI systems do not inherently consider the possible consequences of a misdiagnosis when making a decision. The inability of an AI system to practise with caution highlights the need for clinical validation to prevent harm
Patient access to AI programmes for self‐diagnosis	AI systems may benefit lower income countries. Their possible benefit is limited by data set‐related biases, resource availability and language

## 3 CONCLUSION

There are multiple ethical considerations when implementing AI systems in medicine and dentistry. While there is a general consensus that AI should be ‘ethical’, there is much debate about what ‘ethical’ involves, and hence the essential ethical criteria, technological standards and best practices for AI deployment in medicine and dentistry. Both individuals involved in the creation and deployment of AI systems, and the AI users themselves, bear responsibility for ethical AI usage (Rokhshad et al., 2023). Several key principles of AI use have been suggested (Jobin et al., 2019). Jobin et al. (2019) found global convergence on five key principles of ethical AI use: transparency, fairness and justice, nonmaleficence, responsibility and privacy. The interpretation of each of these principles is unpredictable, leading to variability in how they are ensured when using AI.

Given the ongoing debate surrounding what ethical AI use in dentistry entails, some authors have proposed guidelines to address this issue. For example, Duggal and Tripathi (2024) propose a five‐step ‘RAPID’ technique. This strategy includes (R)egulation, (A)wareness, (P)olicy‐making, (I)mplementation and (E)ducation, all with the goal of promoting and sustaining bioethical values in the use of AI in dentistry (Duggal & Tripathi, 2024). The RAPID technique forms an excellent framework for the ethical implementation of AI in dentistry which can be very easily translated to medicine and healthcare more generally. This model has a strong focus on regulation of AI use, including strict guidelines for assumption of responsibility and clear protocols to maintain ethical standards (Duggal & Tripathi, 2024).

There is great promise for using AI to aid diagnosis within the field of Oral Medicine, particularly in the diagnosis of oral potentially malignant disorders and malignant conditions. This paper has explored the concerns surrounding the use of AI, including potential pitfalls, ethical and workforce implications, which may have serious repercussions in a dental specialty with limited staff capacities and where the cost of misdiagnosis is often severe. The integration of AI into Oral Medicine clinical practice will require careful validation, training and monitoring, as for any other diagnostic tool, to ensure accuracy, safety and effectiveness in supporting healthcare practitioners to deliver care.

## AUTHOR CONTRIBUTIONS

**Qingmei Joy Feng:** Writing – review and editing; writing – original draft. **Molly Harte:** Writing – review and editing; writing – original draft. **Barbara Carey:** Writing – review and editing. **Ali Alqarni:** Writing – review and editing. **Luis Monteiro:** Writing – review and editing. **Márcio Diniz‐Freitas:** Writing – review and editing. **Jean‐Cristophe Fricain:** Writing – review and editing. **Giovanni Lodi:** Writing – review and editing. **Vlaho Brailo:** Writing – review and editing. **Mattia Andreoletti:** Writing – review and editing. **Rui Albuquerque:** Conceptualization; writing – review and editing; supervision.

## FUNDING INFORMATION

No funding was received for this article.

## CONFLICT OF INTEREST STATEMENT

The authors do not have any conflict of interest to declare.

## Data Availability

Data sharing not applicable to this article as no datasets were generated or analysed during the current study.

## References

[odi15100-bib-0001] Adeoye, J. , & Su, Y. X. (2023). Artificial intelligence in salivary biomarker discovery and validation for oral diseases. Oral Diseases, 30(1), 23–37. 10.1111/odi.14641 37335832

[odi15100-bib-0002] Ahn, J. S. , Shin, S. , Yang, S. A. , Park, E. K. , Kim, K. H. , Cho, S. I. , Ock, C. Y. , & Kim, S. (2023). Artificial intelligence in breast cancer diagnosis and personalized medicine. Journal of Breast Cancer, 26(5), 405–435. 10.4048/jbc.2023.26.e45 37926067 PMC10625863

[odi15100-bib-0003] Andreoletti, M. , Haller, L. , Vayena, E. , & Blasimme, A. (2024). Mapping the ethical landscape of digital biomarkers: A scoping review. PLOS Digital Health, 3(5), e0000519. 10.1371/journal.pdig.0000519 38753605 PMC11098308

[odi15100-bib-0004] Bagchi, S. (2023). What is a black box? A computer scientist explains what it means when the inner workings of AIs are hidden. The Conversation.

[odi15100-bib-0005] Chakraborty, D. , Natarajan, C. , & Mukherjee, A. (2019). Advances in oral cancer detection. Advances in Clinical Chemistry, 91, 181–200. 10.1016/bs.acc.2019.03.006 31331489

[odi15100-bib-0006] Challen, R. , Denny, J. , Pitt, M. , Gompels, L. , Edwards, T. , & Tsaneva‐Atanasova, K. (2019). Artificial intelligence, bias and clinical safety. BMJ Quality and Safety, 28(3), 231–237. 10.1136/bmjqs-2018-008370 PMC6560460 30636200

[odi15100-bib-0007] Craig, L. , Laskowski, N. , & Tucci, L. (2024). What is artificial intelligence (AI)? Everything you need to know. TechTarget.

[odi15100-bib-0008] Diniz‐Freitas, M. , Rivas‐Mundiña, B. , García‐Iglesias, J. R. , García‐Mato, E. , & Diz‐Dios, P. (2023). How ChatGPT performs in Oral Medicine: The case of oral potentially malignant disorders. Oral Diseases, 30(4), 1912–1918. 10.1111/odi.14750 37794649

[odi15100-bib-0009] Duggal, I. , & Tripathi, T. (2024). Ethical principles in dental healthcare: Relevance in the current technological era of artificial intelligence. Journal of Oral Biology and Craniofacial Research, 14(3), 317–321. 10.1016/j.jobcr.2024.04.003 38645705 PMC11031811

[odi15100-bib-0010] Esteva, A. , Kuprel, B. , Novoa, R. A. , Ko, J. , Swetter, S. M. , Blau, H. M. , & Thrun, S. (2017). Dermatologist‐level classification of skin cancer with deep neural networks. Nature, 542(7639), 115–118. 10.1038/nature21056 28117445 PMC8382232

[odi15100-bib-0011] Finlayson, S. G. , Bowers, J. D. , Ito, J. , Zittrain, J. L. , Beam, A. L. , & Kohane, I. S. (2019). Adversarial attacks on medical machine learning. Science, 363(6433), 1287–1289. 10.1126/science.aaw4399 30898923 PMC7657648

[odi15100-bib-0012] Gandhi, Z. , Gurram, P. , Amgai, B. , Lekkala, S. P. , Lokhandwala, A. , Manne, S. , Mohammed, A. , Koshiya, H. , Dewaswala, N. , Desai, R. , Bhopalwala, H. , Ganti, S. , & Surani, S. (2023). Artificial intelligence and lung cancer: Impact on improving patient outcomes. Cancers, 15(21), 5236. 10.3390/cancers15215236 37958411 PMC10650618

[odi15100-bib-0013] Higgins, S. , Nazemi, A. , Chow, M. , & Wysong, A. (2018). Review of nonmelanoma skin cancer in African Americans, Hispanics, and Asians. Dermatologic Surgery, 44(7), 903–910. 10.1097/DSS.0000000000001547 29746428

[odi15100-bib-0014] Jobin, A. , Ienca, M. , & Vayena, E. (2019). The global landscape of AI ethics guidelines. Nature Machine Intelligence, 1, 389–399. 10.1038/s42256-019-0088-2

[odi15100-bib-0015] Leslie, D. , Mazumder, A. , Peppin, A. , Wolters, M. K. , & Hagerty, A. (2021). Does “AI” stand for augmenting inequality in the era of covid‐19 healthcare? The British Medical Journal, 372, 304. 10.1136/bmj.n304 PMC7958301 33722847

[odi15100-bib-0016] Mahmood, H. , Shaban, M. , Indave, B. I. , Santos‐Silva, A. R. , Rajpoot, N. , & Khurram, S. A. (2020). Use of artificial intelligence in diagnosis of head and neck precancerous and cancerous lesions: A systematic review. Oral Oncology, 110, 104885. 10.1016/j.oraloncology.2020.104885 32674040

[odi15100-bib-0017] Manning, C. (2020). Artificial intelligence definitiations. Stanford University Human‐Centred Artificial Intelligence.

[odi15100-bib-0018] McKinsey & Company . (2023). What is AI (artificial intelligence)? McKinsey & Company.

[odi15100-bib-0019] Megler, V. , & Gregoire, S. (2018). Training models with unequal economic error costs using Amazon Sagemaker. Amazon web Services.

[odi15100-bib-0020] Melarkode, N. , Srinivasan, K. , Qaisar, S. M. , & Plawiak, P. (2023). AI‐powered diagnosis of skin cancer: A contemporary review, open challenges and future research directions. Cancers, 15(4), 1183. 10.3390/cancers15041183 36831525 PMC9953963

[odi15100-bib-0021] Mittelstadt, B. , & Floridi, L. (2016). The ethics of big data: Current and foreseeable issues in biomedical contexts. Science and Engineering Ethics, 22(2), 303–341. 10.1007/s11948-015-9652-2 26002496

[odi15100-bib-0022] Moharrami, M. , Farmer, J. , Singhal, S. , Watson, E. , Glogauer, M. , Johnson, A. E. W. , Schwendicke, F. , & Quinonez, C. (2024). Detecting dental caries on oral photographs using artificial intelligence: A systematic review. Oral Diseases, 30(4), 1765–1783. 10.1111/odi.14659 37392423

[odi15100-bib-0023] Mörch, C. M. , Atsu, S. , Cai, W. , Li, X. , Madathil, S. A. , Liu, X. , & Ducret, M. (2021). Artificial intelligence and ethics in dentistry: A scoping review. Journal of Dental Research, 100(13), 1452–1460. 10.1177/00220345211013808 34060359

[odi15100-bib-0024] Müller, S. (2022). Is there a civic duty to support medical AI development by sharing electronic health records? BMC Medical Ethics, 23(1), 134. 10.1186/s12910-022-00871-z 36496427 PMC9736708

[odi15100-bib-0025] Nguyen, T. T. , Larrivée, N. , Lee, A. , Bilaniuk, O. , & Durand, R. (2021). Use of artificial intelligence in dentistry: Current clinical trends and research advances. Journal of the Canadian Dental Association, 87(l7), 1488–2159. 34343070

[odi15100-bib-0026] Packhäuser, K. , Gündel, S. , Münster, N. , Syben, C. , Christlein, V. , & Maier, A. (2022). Deep learning‐based patient re‐identification is able to exploit the biometric nature of medical chest X‐ray data. Scientific Reports, 12(1), 14851. 10.1038/s41598-022-19045-3 36050406 PMC9434540

[odi15100-bib-0027] Park, S. H. , Choi, J. , & Byeon, J. S. (2021). Key principles of clinical validation, device approval, and insurance coverage decisions of artificial intelligence. Korean Journal of Radiology, 22(3), 442–453. 10.3348/kjr.2021.0048 33629545 PMC7909857

[odi15100-bib-0028] Rokhshad, R. , Ducret, M. , Chaurasia, A. , Karteva, T. , Radenkovic, M. , Roganovic, J. , & Schwendicke, F. (2023). Ethical considerations on artificial intelligence in dentistry: A framework and checklist. Journal of Dentistry, 135, 104593. 10.1016/j.jdent.2023.104593 37355089

[odi15100-bib-0029] Rokhshad, R. , Mohammad‐Rahimi, H. , Price, J. B. , Shoorgashti, R. , Abbasiparashkouh, Z. , Esmaeili, M. , & Schwendicke, F. (2024). Artificial intelligence for classification and detection of oral mucosa lesions on photographs: A systematic review and meta‐analysis. Clinical Oral Investigations, 28(1), 88. 10.1007/s00784-023-05475-4 38217733

[odi15100-bib-0030] Ruyter, K. W. , Lõuk, K. , Jorqui, M. , Kvalheim, V. , Cekanauskaite, A. , & Townend, D. (2010). From research exemption to research norm: Recognising an alternative to consent for large scale biobank research. Medical Law International, 10(4), 287–313. 10.1177/096853321001000403

[odi15100-bib-0031] Santana, L. C. L. (2023). Artificial Intelligence (AI) and periodontal risk assessment. Oral Diseases, 20, 1–2. 10.1111/odi.14643 37282783

[odi15100-bib-0032] Vayena, E. , Blasimme, A. , & Cohen, I. G. (2018). Machine learning in medicine: Addressing ethical challenges. PLoS Medicine, 15(11), e1002689. 10.1371/journal.pmed.1002689 30399149 PMC6219763

[odi15100-bib-0033] Wahl, B. , Cossy‐Gantner, A. , Germann, S. , & Schwalbe, N. R. (2018). Artificial intelligence (AI) and global health: How can AI contribute to health in resource‐poor settings? BMJ Global Health, 3(4), e000798. 10.1136/bmjgh-2018-000798 PMC6135465 30233828

[odi15100-bib-0034] Willingham, M. L., Jr. , Spencer, S. Y. , Lum, C. A. , Sanchez, J. M. , Burnett, T. , Shepherd, J. , & Cassel, K. (2021). The potential of using artificial intelligence to improve skin cancer diagnoses in Hawaii's multiethnic population. Melanoma Research, 31(6), 504–514. 10.1097/CMR.0000000000000779 34744150 PMC8580213

[odi15100-bib-0035] Zhang, J. , & Zhang, Z. M. (2023). Ethics and governance of trustworthy medical artificial intelligence. BMC Medical Informatics and Decision Making, 23(1), 7. 10.1186/s12911-023-02103-9 36639799 PMC9840286

